# Dataset on passenger experience after riding an autonomous ferry

**DOI:** 10.1016/j.dib.2025.111369

**Published:** 2025-02-03

**Authors:** Erik Veitch, Taufik Akbar Sitompul, Ole Andreas Alsos

**Affiliations:** Department of Design, Norwegian University of Science and Technology (NTNU), Trondheim, Norway

**Keywords:** Questionnaire, Survey, User experience, Safety, Trust, Autonomous vehicle, Autonomous ferry

## Abstract

Autonomous vehicles are being increasingly tested for transportation applications, yet investigations of user experience remain relatively rare. This paper presents a dataset of questionnaire responses (N = 146) investigating user experience of passengers on board an autonomous ferry in Norway. The ferry was the “milliAmpere2,” an autonomous urban ferry owned and operated by the Norwegian University of Science and Technology (NTNU). The dataset consisted of 46 question items across four categories: (i) demographics and background information, (ii) passenger experience, (iii) passenger attitudes, and (iv) feedback on the ferry's design characteristics. Responses were provided voluntarily using a digital survey tool during free-of-charge public trials held between 25 June and 24 August 2024. The dataset provides empirical data on passengers’ experience on board an autonomous ferry. This contributes to design, engineering, urban planning, and social science research related to the use and adoption of autonomous vehicles in public transportation.

Specifications Table

**Table:** 

Subject	Social Science, Safety Science
Specific subject area	User experience of autonomous vehicles.
Data format	Dataset in .csv format (comma-separated text) and questionnaire in PDF/A format.
Type of data	.csv file (dataset containing questionnaire responses) .pdf file (questionnaire in English and Norwegian)
Data collection	A survey was distributed and administered digitally via the survey portal *nettskjema.no*, which is developed and hosted by the University of Oslo (nettskjema@usit.uio.no). The survey was accessible via mobile, tablet, or personal computer in Norwegian and English. Respondents (N = 146) voluntarily accessed and filled out the digital survey via QR code or hyperlink provided on a poster located on the autonomous ferry (convenience sampling). Respondents were entered into a draw for a gift card against voluntary entry of email address (any email address provided have been removed from the dataset). Question items in the survey were derived from twelve studies that investigated passenger experience after using autonomous vehicles in real-world settings. The data collection period was from 25 June to 24 August 2024. Original copies of the survey (in both English and Norwegian) are available in the data repository.
Data source location	Trondheim, Norway (63.434106N, 10.392926S)
Data accessibility	Repository name: DataverseNO Data identification number: 10.18710/9BBPGG The archive is supported by the Norwegian University of Science and Technology (NTNU) and is hosted by UiT The Arctic University of Norway.

## 1 Value of the Data

• The most comprehensive survey of user experience of autonomous ferries undertaken to date, since there are 46 questions covering four categories: (i) demographics and background information, (ii) passenger experience, (iii) passenger attitudes, and (iv) feedback on ferry's design characteristics.
• As the survey contains diverse topics, this dataset may benefit researchers from multiple fields, such as social science, computer science, design, engineering, business, and urban planning.
• These data are useful in understanding the user experience of autonomous ferries as the technology evolves towards implementation in public transportation.
• These data can also be used as an inspiration for other researchers who plan to conduct similar studies.

## 2 Background

The original motivation behind compiling this data was to make more informed design decisions through insights about passengers’ experience on board an autonomous ferry. Persistent knowledge gaps compelled us to formulate a detailed survey that sought answers to specific questions (e.g., Is it clear what passengers should do in case of an evacuation? Are passengers willing to use the ferry without a safety host on board?). In addition, the survey sought general information about passengers and their preference (e.g., demographics and attitudes towards public transportation). Survey questions were designed based on a literature review of peer-reviewed, survey-based investigations of passenger experience after using autonomous vehicles in real-world settings. A total of twelve studies were reviewed that focused on autonomous cars [[1], [2], [3]], buses [[4], [5], [6], [7]], and shuttles [[8], [9], [10], [11], [12]]. The reader is advised to see Table 5, Table 6, and Table 7 to find the specific information about which studies were used to develop the survey questions.

**Fig. 1:**
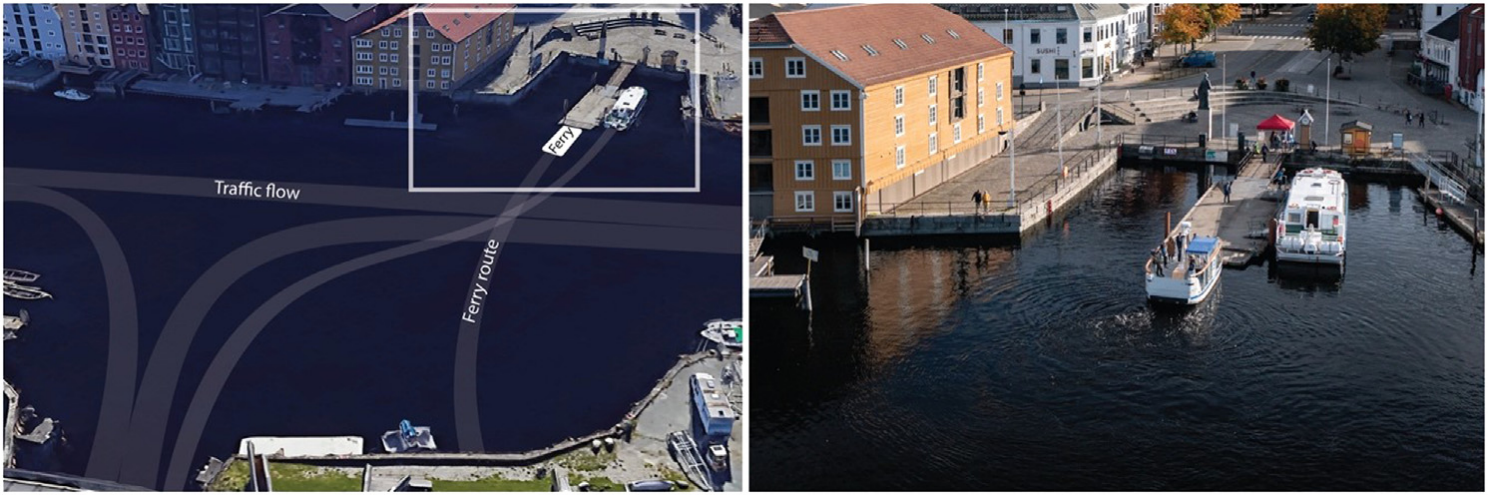
The “milliAmpere2” autonomous ferry during a public trial in Trondheim, Norway (Left: Photo credit Google Maps; Right: Photo credit Mikael Sætereid).

The ferry featured in this study was the “milliAmpere2,” which is an 8.6-meter-long autonomous urban passenger ferry owned and operated by NTNU (see Fig. 1; see [13] for details). A survey-based dataset was also generated in 2022 using the “milliAmpere2,” albeit with a narrow user experience scope [14]. The dataset herein follows up the previous one based on lessons learned [15]. The main differences between the dataset generated in 2022 [14] and the dataset presented in this paper can be found in Table 1.

**Table 1:** The main differences between the dataset generated in 2022 [14] and the dataset described in this paper.

Criteria	The dataset generated in 2022 [14]	The dataset described in this paper
Number of questions	7 questions before the passengers took the ferry and 5 questions after the passengers took the ferry.	46 questions that were filled after the passengers took the ferry
Data collection	The data were collected twice: before and after taking the ferry. There were also on-site personnel administering the surveys to the passengers	The data were collected after taking the ferry only. The data collection was done voluntarily by passengers without the presence of any on-site personnel.
Topics in the survey	Trust and safety perceptions before and after taking the ferry	Passenger experience after taking the ferry, passenger attitudes towards the ferry, and feedback on the ferry's design characteristics.

## 3 Data Description

The questionnaire used to generate the dataset is available in the DataverseNO repository as a PDF/A file [16]. Both the English version (*“Questionnaire-English.pdf”*) and Norwegian version (*“Questionnaire-Norwegian.pdf”*) are available*.* All respondents completed the survey individually and immediately after using the autonomous ferry (N = 146). Responses are available as comma-separate text in “*dataset-25,062,024-to-24,082,024.csv.*” This dataset merges responses from Norwegian and English questionnaires into a single English translation. The responses are labeled and coded according to the four tables listed in Appendix A.

Basic demographics of the sample are listed in Table 2, with additional information (e.g., education, employment status, place of residence) available in the dataset. Although the sample age structure was generally representative of the population, the age group 20–29 was overrepresented by approximately 13.5 % (see Table 3). To avoid introducing age-related biases into the findings, Table 3 can be used to generate weights for analyses. Weights can be computed by dividing each age group's population proportion by the corresponding sample proportion (e.g., Weight_(20–29 years)_ = 18/31.5 = 0.57). Weights can then be applied as a multiplying factor to the data in each corresponding age group. Only individuals 18 years and older are included in the dataset.

**Table 2:** Descriptive statistics of sample demographics (N = 146).

Age	
Mean (SD)	41 (15)
Mode	25
Minimum	20
25th percentile	27
Median	40
75th percentile	52
Maximum	82
Gender	

Male	80
Female	65
Other	1

**Table 3:** Age structure for respondents and for population.

Age group	Sample	Population*	Sample (%)	Population (%)
0–9 years	0	21 563	0.0 %	10.0 %
10–19 years	0	23 786	0.0 %	11.1 %
20–29 years	46	38 674	31.5 %	18.0 %
30–39 years	26	33 575	17.8 %	15.6 %
40–49 years	27	27 124	18.5 %	12.6 %
50–59 years	27	25 773	18.5 %	12.0 %
60–69 years	16	20 359	11.0 %	9.5 %
70–79 years	3	15 689	2.1 %	7.3 %
80–89 years	1	6 664	0.7 %	3.1 %
90–99 years	0	1 331	0.0 %	0.6 %
100 years and older	0	27	0.0 %	0.0 %
*Total*	*146*	*214,565*		

⁎ Population of Trondheim municipality in Q2 of 2024 [17].

## 4 Experimental Design, Materials and Methods

A passenger survey campaign was conducted during a public trial of the “milliAmpere2,” held in Trondheim, Norway, from 25 June to 24 August 2024. The survey consisted of a questionnaire containing 46 questions, available in Norwegian and English. The questionnaire was distributed and administered digitally via the survey portal *nettskjema.no*, which is developed and hosted by the University of Oslo. Respondents voluntarily accessed and filled out the questionnaire via a QR code or hyperlink provided on a poster located on a ferry (convenience sampling). The median time to complete the questionnaire was approximately 7 min. Respondents were entered into a draw for a gift card against voluntary entry of email address (email addresses were removed from the dataset). The dataset does not contain identifiable personal data. A total of approximately 2300 passengers used the ferry during the trial, of which 146 individuals 18 years or older filled out the questionnaire. During the trial, the ferry crossed a 100-m canal and anyone could ride the ferry free of charge. Safety operators were on board during all crossings. Safety operators were individuals with the necessary certificates for operating the ferry, as prescribed by the Norwegian Maritime Authority (see [13] for details).

Appendix A lists all question items across four relevant categories: (i) demographics and background information (8 questions; Table 4), (ii) passenger experience (17 questions; Table 5), (iii) passenger attitudes (9 questions; Table 6), and (iv) feedback on the ferry's design characteristics (12 questions; Table 7).

## Limitations

Some limitations stem from the sampling protocol and sample statistics. Specifically, convenience sampling may have resulted in an optimism bias because respondents were individuals who were willing to use the ferry in the first place (or perhaps actively sought it out). By comparison, a more representative sampling protocol could have selected individuals randomly and invited them to use the ferry. In addition, a relatively small sample size was collected over the two-month trial period (N = 146) compared to the potential user base. Moreover, overrepresentation of the age group 20–29 may also have introduced bias, which should be accounted for in any post-hoc analysis.

## Ethics Statement

This study was audited and approved by the Norwegian Agency for Shared Services in Education and Research (Sikt), which oversees the ethical conduct and participant privacy of research at Norwegian institutions. The data collection and management plan described herein were assigned the Sikt project number 340097. The main measures in place to comply with Sikt's ethical research standards included informed consent and anonymity. Informed consent was obtained from all participants in the study. Anonymity was maintained by assigning participants numeric codes and by removing potentially de-anonymizing data (e.g., email addresses) from the dataset.

## CRediT Author Statement

**Erik Veitch**: Writing—Original Draft, Conceptualization, Investigation. **Taufik Akbar Sitompul**: Methodology, Investigation, Writing—Review and Editing. **Ole Andreas Alsos**: Writing—Review and Editing, Supervision.

## Figures and Tables

**Table 4:** Dataset labels for Category 1 (Demographics and background information).

Label	Description
submission_id	Anonymized identifier
Created	Date and time submission created
answer_time_ms	Time to complete survey (milliseconds)
Age*	Age
1 - What is your gender?	Man, Woman, Other
2 - What is your highest educational level?	Basic Education, Upper Secondary School, Bachelor, Master, PhD
3 - What is your employment status?	Unemployed, Student, Part-time, Full-time, Retired
4 - Where do you live?	Trondheim, Elsewhere in Trøndelag, Elsewhere in Norway, Outside Norway
4B - Which country do you live in?	Entered only if “Outside Norway” is selected for question above.
5 - What is your usual mode of transportation? (Can choose multiple)	Walking, Cycling, E-Scooter, Bus, Train, Car, Ferry
6 - How frequently do you use public transportation in a week?	Daily, More that 3 times a week, 1 to 3 times a week, Less than once a week, Never
7 - With whom will you take the autonomous ferry?	Alone, With friends, With family, With colleagues
8 - Have you taken the autonomous ferry before?	Yes, No

⁎ This dataset has been filtered to remove all respondents under the age of 18.

**Table 5:** Dataset labels for Category 2 (Passenger experience).

Label	Description	Adapted from
9 - I feel very safe inside the autonomous ferry throughout the whole trip.	1 = Strongly disagree; 3 = Neutral; 5 = Strongly agree	[3]
10 - In general, it is easy to use the autonomous ferry.	1 = Strongly disagree; 3 = Neutral; 5 = Strongly agree	[4,10]
11 - I do not need to take special training to use the autonomous ferry.	1 = Strongly disagree; 3 = Neutral; 5 = Strongly agree	[2]
12 - The information provided before riding the autonomous ferry is clear and understandable.	1 = Strongly disagree; 3 = Neutral; 5 = Strongly agree	[10]
13 - The information provided on board the autonomous ferry is clear and understandable.	1 = Strongly disagree; 3 = Neutral; 5 = Strongly agree	
14 - The information provided after riding the autonomous ferry is clear and understandable.	1 = Strongly disagree; 3 = Neutral; 5 = Strongly agree	
15 - I feel very safe riding the autonomous ferry.	1 = Strongly disagree; 3 = Neutral; 5 = Strongly agree	[3,6]
16 - The speed of the autonomous ferry is.	1 = Too slow; 3 = Just right; 5 = Too fast	[3,7,11]
17 - The way the autonomous ferry brakes is.	1 = Too careful; 3 = Just right; 5 = Too sudden	[7]
18 - The autonomous ferry is safe enough (in terms of technical failures and accidents).	1 = Strongly disagree; 3 = Neutral; 5 = Strongly agree	[5]
19 - The autonomous ferry is safe enough (in terms of crimes).	1 = Strongly disagree; 3 = Neutral; 5 = Strongly agree	
20 - It is clear how I should evacuate the ferry in case of an emergency.	1 = Strongly disagree; 3 = Neutral; 5 = Strongly agree	[9]
21 - The autonomous ferry is environmentally friendly.	1 = Strongly disagree; 3 = Neutral; 5 = Strongly agree	[5]
22 - I have concerns about the data that the autonomous ferry collects about me (e.g., video camera)	1 = Strongly disagree; 3 = Neutral; 5 = Strongly agree	
23 - I am satisfied with the overall condition on board the autonomous ferry.	1 = Strongly disagree; 3 = Neutral; 5 = Strongly agree	[4]
24 - I am satisfied with the overall condition of the piers on both sides.	1 = Strongly disagree; 3 = Neutral; 5 = Strongly agree	
25 - I am satisfied with the waiting time at the piers.	1 = Strongly disagree; 3 = Neutral; 5 = Strongly agree	

**Table 6:** Dataset labels for Category 3 (Passenger attitudes).

Label	Description	Adapted from
26 - I find that riding the autonomous ferry to cross the canal is more convenient than walking over the nearest bridges.	1 = Strongly disagree; 3 = Neutral; 5 = Strongly agree	[4,11]
27 - I find that riding the autonomous ferry to cross the canal is more convenient than cycling over the nearest bridges.	1 = Strongly disagree; 3 = Neutral; 5 = Strongly agree	
28 - The autonomous ferry is an efficient mode of transportation across the canal.	1 = Strongly disagree; 3 = Neutral; 5 = Strongly agree	
29 - I am willing to use the autonomous ferry regularly if the service is offered in the future.	1 = Strongly disagree; 3 = Neutral; 5 = Strongly agree	[3,4,6]
30 - I strongly recommend that other people use the autonomous ferry.	1 = Strongly disagree; 3 = Neutral; 5 = Strongly agree	[4]
31 - The autonomous ferry will be an important part of public transportation in Trondheim.	1 = Strongly disagree; 3 = Neutral; 5 = Strongly agree	[7,11]
32 - How long would you be willing to wait for the autonomous ferry?	1–3 min, 4–6 min, 7–10 min, More than 10 min	[3]
33 - How much would you be willing to pay every time to ride the autonomous ferry? (prices in NOK)	5–10 kr, 11–20 kr, 31–30 kr, More than 30 kr	[3,4,11]
34 - Should the ticket for the autonomous ferry service be included in the AtB's* period tickets?	Yes, No	[11]

⁎ AtB is the municipal transportation provider in Trondheim, operating local buses, ferries, and city bikes.

**Table 7:** Dataset labels for Category 4 (Feedback on the ferry's design characteristics).

Label	Description	Adapted from
35 - The size of the autonomous ferry is adequate.	1 = Strongly disagree; 3 = Neutral; 5 = Strongly agree	[11]
36 - It is easy to board and disembark the autonomous ferry.	1 = Strongly disagree; 3 = Neutral; 5 = Strongly agree	[3]
37 - There is enough seating on the autonomous ferry.	1 = Strongly disagree; 3 = Neutral; 5 = Strongly agree	[11]
38 - The seating area on the autonomous ferry is comfortable.	1 = Strongly disagree; 3 = Neutral; 5 = Strongly agree	
39 - There is enough space for standing on the autonomous ferry.	1 = Strongly disagree; 3 = Neutral; 5 = Strongly agree	
40 - There is enough space for luggage, strollers, and bicycles on the autonomous ferry.	1 = Strongly disagree; 3 = Neutral; 5 = Strongly agree	
41 - The autonomous ferry should provide sufficient coverage for bad weather conditions.	1 = Strongly disagree; 3 = Neutral; 5 = Strongly agree	
42 - I am willing to use the autonomous ferry without a safety host on board.	1 = Strongly disagree; 3 = Neutral; 5 = Strongly agree	[5,11]
43 - A safety host on board is important for maintaining security of the autonomous ferry (assaults, robberies, etc.).	1 = Strongly disagree; 3 = Neutral; 5 = Strongly agree	[5]
44 - A safety host on board is important for maintaining security of the autonomous ferry (technical failures, accidents, etc.).	1 = Strongly disagree; 3 = Neutral; 5 = Strongly agree	
45 - A safety host on board is important for maintaining good passenger experience (boarding/deboarding, providing information, etc.).	1 = Strongly disagree; 3 = Neutral; 5 = Strongly agree	
46 - I am not worried about the safety of other canal users (other vessels, boats, kayaks, etc.).	1 = Strongly disagree; 3 = Neutral; 5 = Strongly agree	[8]

## Data Availability

DataverseQuestionnaire dataset about user experience of an autonomous ferry (Original data).
